# Comparison of Intradermal Versus Microneedling‐Assisted Botulinum A Toxin Injection for Enlarged Facial Pores: A Randomized Clinical Trial

**DOI:** 10.1111/jocd.70114

**Published:** 2025-06-16

**Authors:** Fariba Iraji, Reza Moeini, Mahya Abedini, Marzieh Sadat Mousavi, Mina Saber, Mahmoud Reza Rahimi Barghani, Malihe Sagheb Ray Shirazi, Sarah Seyedyousefi

**Affiliations:** ^1^ Skin Diseases and Leishmaniasis Research Center, Department of Dermatology Isfahan University of Medical Sciences Isfahan Iran; ^2^ Isfahan University of Medical Sciences Isfahan Iran; ^3^ Department of Anatomical Sciences, Faculty of Nursing and Midwifery Hormozgan University of Medical Sciences Bandar Abbas Iran; ^4^ Skin Diseases and Leishmaniasis Research Center Isfahan University of Medical Sciences Isfahan Iran

**Keywords:** botulinum toxins, cosmetic techniques, intradermal injections, microneedling, skin pores, type A

## Abstract

**Background:**

Enlarged facial pores are a prevalent cosmetic concern affecting many individuals. Traditional treatments include topical agents and laser therapies, but recent advancements have introduced intradermal and microneedling‐assisted Botulinum toxin type A (BoNTA) injections as promising alternatives. This study aims to compare the efficacy of these two methods in reducing the size of enlarged facial pores.

**Methods:**

This randomized clinical trial was conducted at a referral centre in Iran. Thirty patients aged 25–60 years with enlarged facial pores were enrolled. Patients were randomized to receive intradermal BoNTA injections on one side of the face and microneedling‐assisted BoNTA injections on the other side. Dermoscopic evaluation and physical examination were performed at baseline and 1‐month post‐treatment. Improvement in pore size was assessed by three blinded dermatologists using the Quartile Improvement Scale (QIS), and patient satisfaction was measured using the Likert scale.

**Results:**

The average age of participants was 34.2 years, with 29 females and one male. Dermoscopic and physical examination scores showed no significant difference between the two treatment modalities for both cheek and nose areas (*p* > 0.05). Patient satisfaction scores were also comparable between the two sides (*p* = 0.13). Both treatments effectively reduced pore size, but no statistically significant difference was observed between intradermal and microneedling‐assisted BoNTA injections.

**Conclusion:**

Both intradermal and microneedling‐assisted BoNTA injections are effective in treating enlarged facial pores, with no significant difference in efficacy. These findings provide flexibility for dermatologists in selecting the appropriate treatment method based on patient preferences and clinical considerations.

## 1 Introduction

Enlarged facial pores are a common cosmetic concern that can significantly impact an individual's self‐esteem and skin appearance. These pores are often associated with increased sebum production and skin laxity, prevalent conditions among individuals with oily skin and those suffering from facial seborrhea. Managing enlarged facial pores has traditionally involved various dermatological treatments, including topical agents, chemical peels, and laser therapies. However, recent advancements have introduced novel approaches, such as intradermal and microneedling‐assisted botulinum toxin type A (BoNTA) injections. These have shown promising results in improving skin texture and reducing pore size [1, 2, 3].

Intradermal injections of BoNTA have gained popularity due to their efficacy in reducing sebum production and improving the appearance of enlarged facial pores [2]. BoNTA works by inhibiting acetylcholine release at the neuromuscular junction, reducing sebaceous gland activity and sebum production [2]. Clinical studies have demonstrated significant improvements in pore size and skin texture following intradermal BoNTA injections [4]. For instance, a split‐face controlled pilot study involving 20 patients with enlarged facial pores and seborrhea reported a significant reduction in sebum and pore scores on the BoNTA‐treated side compared to the saline‐treated side, with results lasting up to 4 months [2].

Microneedling, also known as collagen induction therapy, involves using fine needles to create micro‐injuries in the skin, stimulating collagen production and enhancing skin rejuvenation [3]. When combined with BoNTA, microneedling can enhance the delivery and efficacy of the toxin. This combination therapy has been shown to improve skin texture, reduce pore size, and decrease sebum production. A study comparing microneedling combined with BoNTA versus microneedling combined with platelet‐rich plasma (PRP) found that both treatments were effective in treating atrophic acne scars, with comparable efficacy in improving skin quality [5].

The comparative efficacy of intradermal versus microneedling‐assisted BoNTA injections for the treatment of enlarged facial pores remains an area of active research. Intradermal BoNTA injections offer a minimally invasive option with a relatively straightforward application process, while microneedling‐assisted BoNTA injections may provide additional benefits through enhanced collagen production and skin rejuvenation. Studies have shown that both methods effectively reduce pore size and improve skin texture, but further research is needed to determine the optimal approach for different patient populations and skin types [4, 6]. In this randomized clinical trial, we aimed to evaluate the Outcomes of patient and physician‐reported outcome measures of intradermal versus microneedling‐assisted BoNTA injection for enlarged facial pores.

## 2 Methods

### 2.1 Study Design

The present study is a randomized clinical trial aimed to investigate the outcomes of intradermal versus microneedling‐assisted Botulinum A toxin injection for patients with enlarged facial pores. Enrollment was carried out in a referral center in Isfahan. The Institutional Review Board (IRB) approved the current study. The study protocol was registered in the Iranian Clinical Trial Registry (IRCT) and was approved with a unique identification number of IRCT20211010052723N2. The study complied with the Declaration of Helsinki.

### 2.2 Inclusion and Exclusion Criteria

Subjects were healthy adult individuals (age range 25–60 years old) with varying degrees of enlarged dilated facial pores. Subjects who received topical or systemic medical treatment, including topical or oral retinoids, within the last 3 months were excluded. In addition, patients who had undergone chemical peels or Intense Pulsed Light Therapy (IPL) within the last 6 months of enrollment were excluded. Other exclusion causes were patients with a known sensitivity to Botulinum A toxin, pregnant or breastfeeding mothers, and patients with a known neuromuscular disorder, including Myasthenia gravis. The sample size was calculated to ensure an 80% power to detect a difference between the groups at a two‐sided significance level of 0.05. It was determined that 35 patients per group would be sufficient to achieve statistical significance.

### 2.3 Interventions and Treatment Process

This study was conducted as a split‐face clinical trial. Initially, eligible patients who provided consent were randomized to receive treatment on one side of their face with botulinum toxin A via intradermal injections while the opposite side underwent microneedling and infiltration of the BoNTA. Following local anesthesia at the treatment sites, botulinum toxin A was prepared at a concentration of 100 units per 2.5 mL and loaded into a one cc syringe equipped with a 30‐gauge needle, with each syringe containing 20 units of the toxin. Subsequently, one side of the face was treated with a 24‐needle microneedling device, penetrating to depths of 3 to 3.5 mm until pinpoint bleeding was observed. Fifty units of the Botulinum toxin solution were then administered to the area.

For the contralateral side, the intradermal injection was performed using pre‐prepared one cc syringes, with injections spaced 1 cm apart into the dermis to induce a slight elevation. Each injection dispensed a portion of the total 50 units of the solution. Post‐treatment, patients were directed to maintain an upright position for a minimum of 4 h and to avoid vigorous physical activities for the next 24 h. They were also counseled to avoid using ibuprofen, aspirin, fish oil, and vitamin E supplements, as well as cosmetic products for 24 h. Furthermore, patients were instructed to stay clear of direct sunlight exposure on the treated area to prevent any potential complications.

### 2.4 Outcome Analysis and Variables

Patient information, including age, location of lesions, previous treatments, and the size of lesions, was recorded. Skin type was assessed as dry, mild, moderate, and very oily. Patients underwent dermoscopic evaluation (Fotofinder Dermlite Handyscope—with 10× magnification) to assess the number and size of pores in a targeted area, focusing on shallow pits and pores filled with keratotic plugs before and 1‐month after treatment. Photographic documentation was carried out at baseline and 1‐month post‐treatment using a Nikon Digital Camera D7100. The improvement in pore size was assessed and quantified by three blinded dermatologists using the Quartile Improvement Scale (QIS). The Likert satisfaction scale was utilized 1‐month after the interventions for a patient‐reported outcome measure, utilizing the following scale: (1) very dissatisfied, (2) dissatisfied, (3) neither satisfied nor dissatisfied, (4) satisfied, and (5) very satisfied.

### 2.5 Statistical Analysis

Data was entered and analyzed via SPSS version 26.0. Descriptive data are reported as mean, standard deviation (SD), frequency, and percentage (%). Inter‐rater agreement between two examiners was assessed using Cohen's kappa statistic. Cross‐tabulation analysis was conducted, followed by computation of Cohen's kappa to determine agreement beyond chance. Because this was a split‐face design in which each participant received both treatments (intradermal vs. microneedling‐assisted BoNTA) on opposite sides of the face, we used a paired t‐test to compare outcomes between the two sides. Prior to conducting the paired t‐test, the distribution of the data was evaluated using the Shapiro–Wilk test to assess normality. Levene's test was also used to check for homogeneity of variance. As our data did not show significant deviations from normality or unequal variances, parametric tests were deemed appropriate. A *p*‐value of less than 0.05 was considered statistically significant.

## 3 Results

Our study evaluated 30 patients. The average age of the patients was 34.2 (range: 22–48; SD: 7.1) years, and 1 was male, while 29 were female. Regarding skin type, 2 (6.7%) were mild, 6 (20.0%) were moderate, 14 (46.7%) were oily, and 8 (26.7%) were very oily.

**FIGURE 1:**
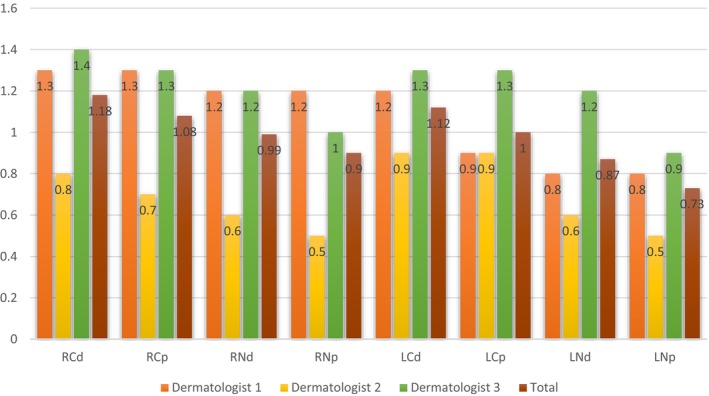
Evaluation of the dermatology and physical examination scores by the dermatologists. LCD, left cheek dermoscopy; LCP, left cheek physical exam; LND, left nose dermoscopy; LNP, left nose physical exam; RCD, right cheek dermoscopy; RCP, right cheek physical exam; RND, right nose dermoscopy; RNP, right nose physical exam.

The average score for right cheek dermoscopy and physical examination was 1.2 ± 0.5 and 1.1 ± 0.5, while for left cheek dermoscopy and physical examination, it was 1.1 ± 0.6 and 1.0 ± 0.5, respectively (*p* = 0.69 and 0.55, respectively). The average score for right nose dermoscopy and physical examination was 1.0 ± 0.6 and 0.9 ± 0.5, while for left nose dermoscopy and physical examination, it was 0.9 ± 0.5 and 0.7 ± 0.5, respectively (*p* = 0.39 and 0.22, respectively). Based on statistical analysis, there was no significant difference between the cheek and nose scores between the two groups. Figure 1 demonstrates the average scores of the patients in our study.

The average patient satisfaction score for the left side was 3.5 ± 0.7, while that for the right side was 3.8 ± 0.8, with no statistically significant difference between the two groups (*p* = 0.13).

The results of patients who underwent intradermal and microneedling‐assisted Botulinum A toxin injection are shown inFigures 2, 3, 4, 5.

**FIGURE 2:**
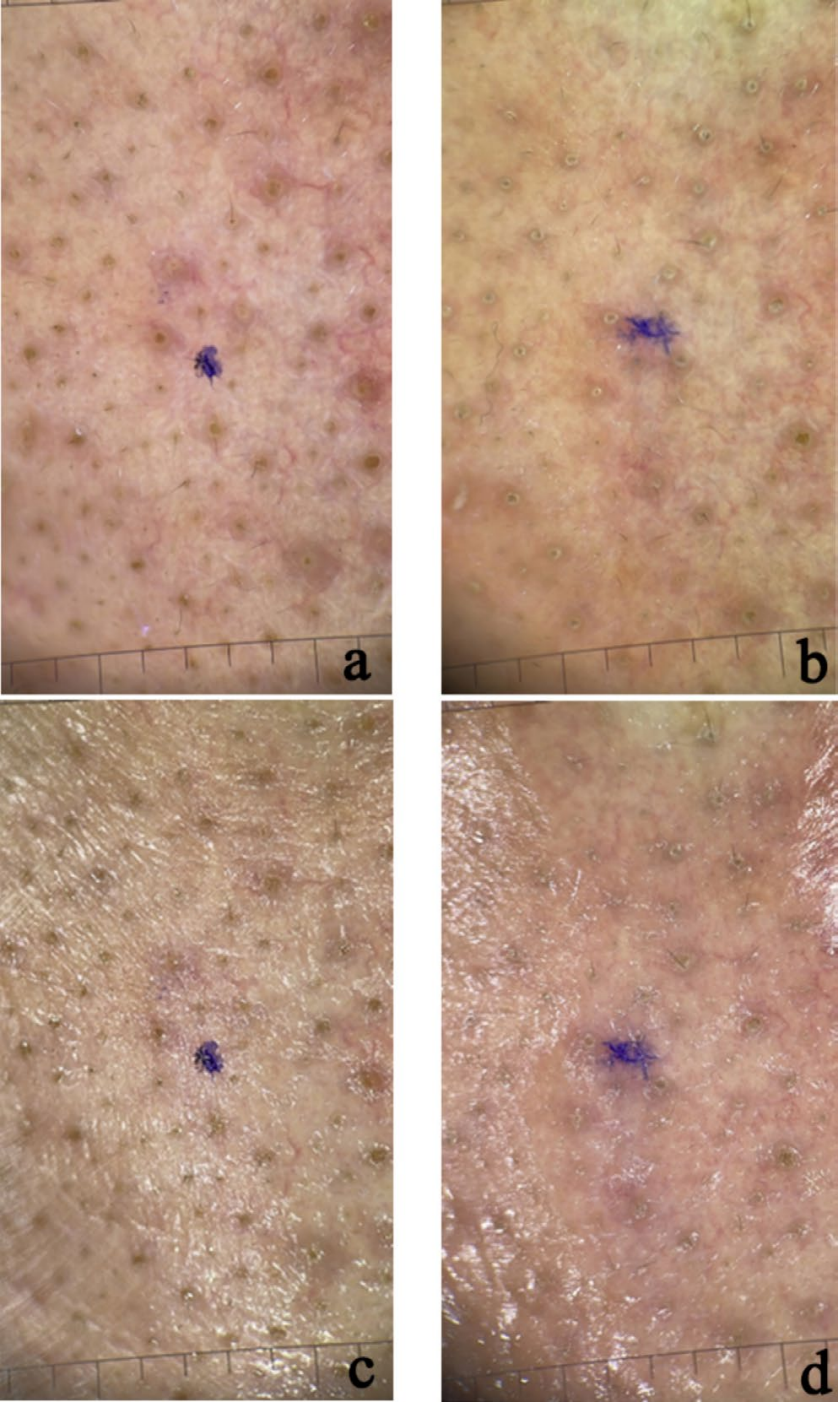
Polarized (a, b) and non‐polarized (c, d) dermoscopic photos of nasal area skin in a patient treated with microneedling and botox injections (a) before treatment—polarized; (b) after a month of treatment—polarized; (c) before treatment—nonpolarized; (d) after a month of treatment—nonpolarized.

**FIGURE 3:**
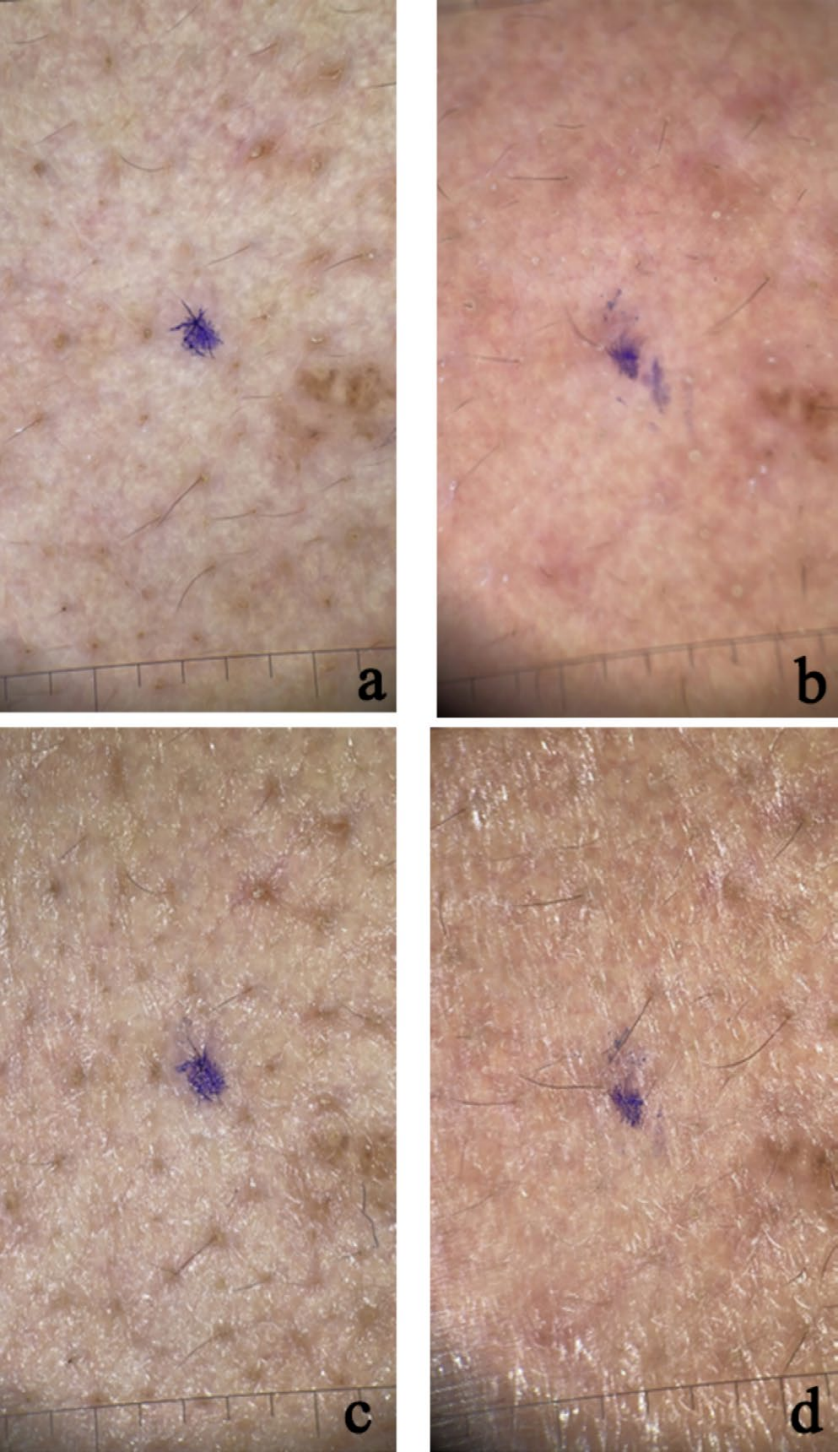
Polarized (a,b) and non‐polarized (c, d) dermoscopic photos of cheek area skin in a patient treated with microneedling and botox injections (a) before treatment—polarized; (b) after a month of treatment—polarized; (c) before treatment—nonpolarized; (d) after a month of treatment—nonpolarized.

**FIGURE 4:**
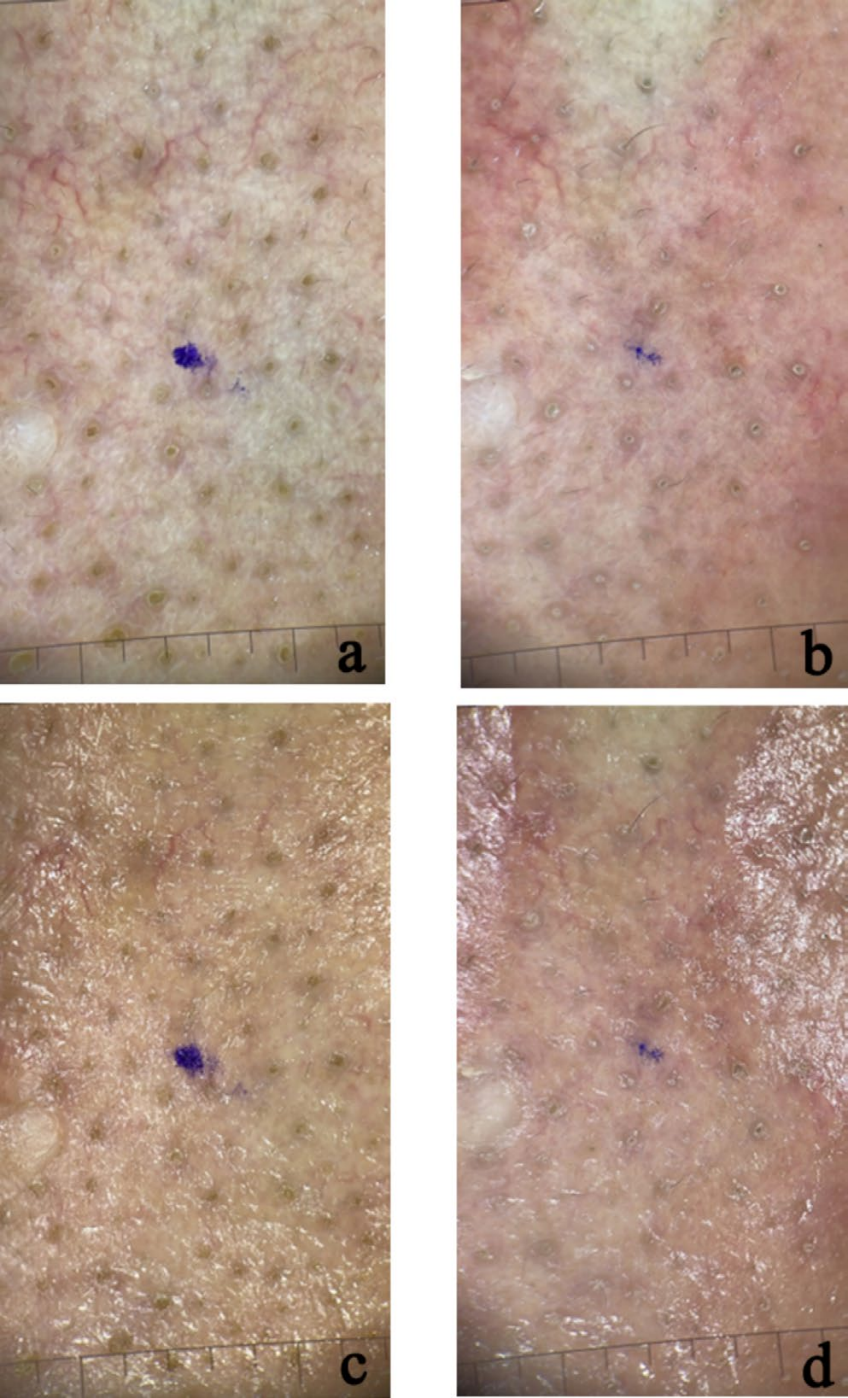
Polarized (a, b) and non‐polarized (c, d) dermoscopic photos of nasal area skin in a patient treated with mesotherapy and botox injections (a) before treatment—polarized; (b) after a month of treatment—polarized; (c) before treatment—nonpolarized; (d) after a month of treatment—nonpolarized.

**FIGURE 5:**
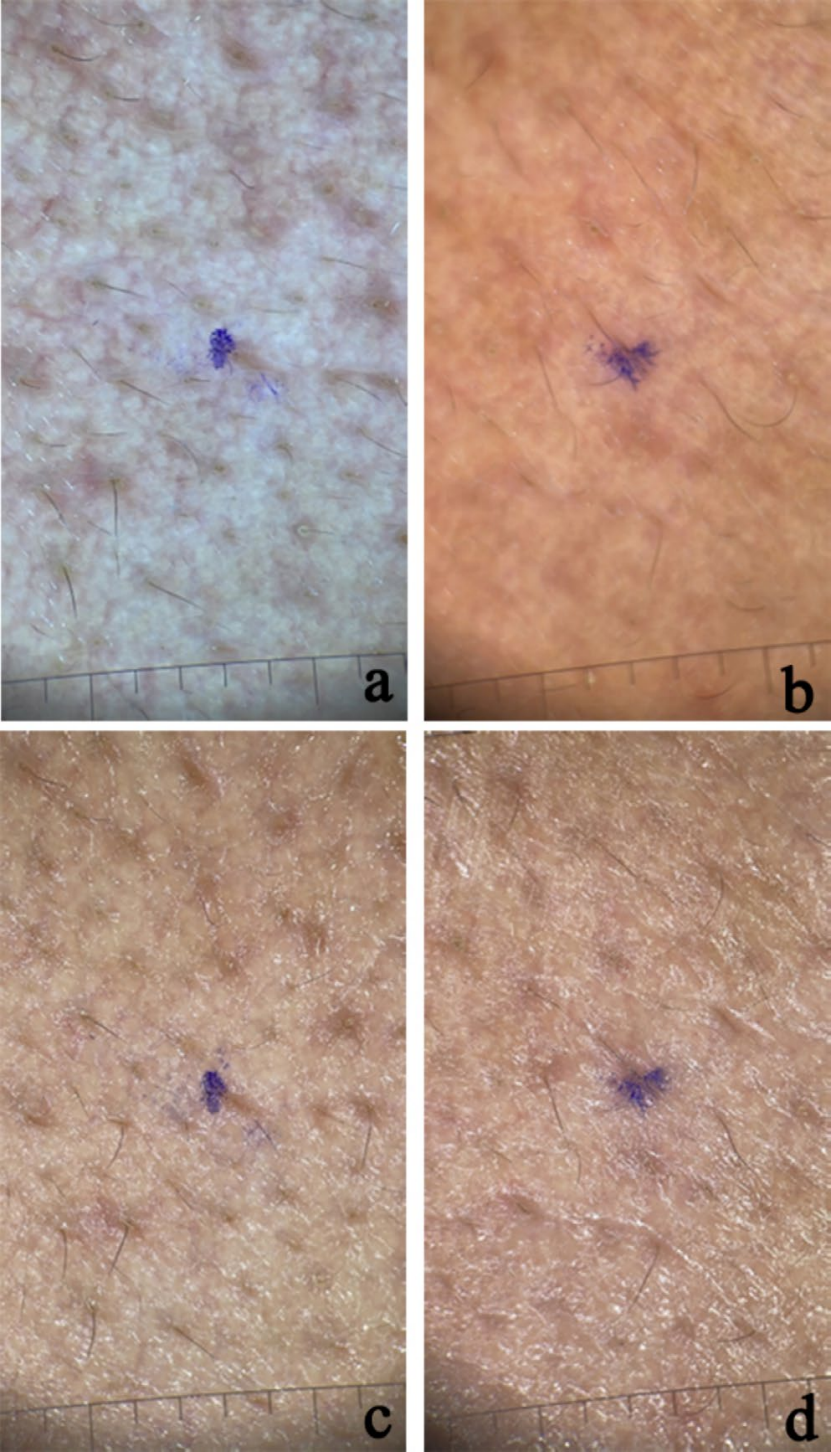
Polarized (a, b) and non‐polarized (c, d) dermoscopic photos of cheek area skin in a patient treated with mesotherapy and botox injections (a) before treatment—polarized; (b) after a month of treatment—polarized; (c) before treatment—nonpolarized; (d) after a month of treatment—nonpolarized.

## 4 Discussion

The present study aimed to compare the efficacy of intradermal versus microneedling‐assisted Botulinum A toxin injection for the treatment of enlarged facial pores. Our results demonstrated no statistically significant difference between the two treatment modalities regarding dermoscopic and physical examination scores for both cheek and nose areas. Additionally, patient satisfaction scores were comparable between the two sides, with no significant difference observed.

These findings are consistent with previous studies that have shown the effectiveness of intradermal Botulinum toxin injections and microneedling‐assisted delivery in improving the appearance of enlarged facial pores. Intradermal Botulinum toxin injections have been reported to reduce pore size and sebum production by inhibiting acetylcholine release at the neuromuscular junction, which in turn affects sebaceous gland activity [3, 7]. The mechanism is thought to involve both direct paralysis of arrector pili muscles and indirect reduction of sebum production [8]. Microneedling, on the other hand, has been shown to enhance the delivery of topically applied substances, including Botulinum toxin, by creating microchannels in the skin [9]. This technique not only facilitates the penetration of the toxin but also stimulates collagen production, potentially leading to additional improvements in skin texture and pore appearance [5].

The comparable efficacy observed in our study between intradermal injection and microneedling‐assisted delivery of Botulinum toxin aligns with findings from other researchers. For instance, a study by Salem et al. comparing microbotox injection to its topical application following microneedling found both methods to be effective in minimizing enlarged facial pores, with intradermal injection showing slightly more patient satisfaction [3].

Our results also corroborate the findings of Ebrahim et al., who reported similar efficacy between microneedling delivery and intradermal injection of Botulinum toxin for facial hyperhidrosis. Microneedling showed higher patient satisfaction due to reduced pain during the procedure [8]. This is particularly relevant as patient comfort and satisfaction are crucial factors in cosmetic procedures.

Our study findings align with previous literature regarding post‐procedural downtime and complications associated with intradermal and microneedling‐assisted BoNTA injections. In our cohort, minimal swelling was observed on the intradermal injection side in some patients, resolving within a maximum of 2 h. A few patients experienced mild erythema on the microneedling‐treated side, which subsided within 24 h as well. No cases of post‐inflammatory hyperpigmentation were recorded. However, it is important to acknowledge that our study did not systematically assess downtime parameters, such as precise erythema duration in minutes or patient‐reported recovery experiences. Previous studies have reported similar trends, with microneedling‐associated erythema typically resolving within 24 to 72 h [1, 5] and intradermal BoNTA injections generally causing only transient swelling that gets better within hours [8]. Future research should include standardized assessments of downtime and recovery timelines to further differentiate the post‐procedural experiences of these two techniques.

The lack of significant difference between the two methods in our study suggests that both techniques can be considered viable options for treating enlarged facial pores. This provides dermatologists with flexibility in choosing the most appropriate method based on individual patient factors, such as pain tolerance, preference for minimally invasive procedures, or specific skin characteristics.

Our study has several limitations that should be considered. Firstly, the sample size was relatively small, which may limit the generalizability of our findings. We acknowledge the relatively small sample size (*n* = 30), which may reduce the power of our statistical comparisons and the generalizability of our results. We therefore agree that future studies should include larger patient cohorts to confirm these findings and should consider consulting a statistician to explore additional methods of analysis (e.g., non‐parametric tests, mixed‐effects models) in cases of non‐normal data or to more rigorously account for the split‐face, repeated‐measures nature of the study. Secondly, the follow‐up period was limited to 1‐month post‐treatment. A longer follow‐up duration would provide valuable insights into the longevity of the treatment effects. Additionally, our study population was predominantly female, which may not fully represent the diverse patient population seeking treatment for enlarged facial pores. Finally, while we used dermoscopic evaluation and physical examination scores, incorporating more objective measurement tools, such as three‐dimensional imaging or sebum production quantification, could provide more precise data on treatment outcomes.

## 5 Conclusion

Our study demonstrates that both intradermal Botulinum toxin injection and microneedling‐assisted delivery are effective in improving the appearance of enlarged facial pores, with no significant difference in efficacy between the two methods. These findings offer valuable insights for dermatologists and patients when selecting treatment options for this common cosmetic concern. Intradermal and microneedling‐assisted BoNTA injections are promising therapeutic options, significantly improving skin texture and pore size through minimally invasive methods. Future studies with larger sample sizes, longer follow‐up periods, and more diverse patient populations are warranted to further elucidate the comparative efficacy and long‐term outcomes of these treatment modalities. Ongoing research and clinical trials will continue to refine these techniques and expand their applications in dermatological practice.

## Author Contributions

F.I. conceptualized the study and designed it. R.M. contributed in the study design and data acquisition. M.A., M.S.M., M.S., M.R.R.B. contributed in data acquisition, data analysis and drafting the manuscript. M.S.R.S. and S.S. contributed in data acquisition and manuscript drafting and editing.

## Ethics Statement

This study was approved by the ethics committee of Isfahan University of Medical Sciences (Code: IR.ARI.MUI.REC.1401.069) and also approved by the registry for clinical trials (Code: IRCT20211010052723N2; registered on 2022‐07‐01, available at: irct.behdasht.gov.ir/trial/64249). The study was conducted in accordance with the relevant guidelines and regulations and the Declaration of Helsinki. Written informed consent is obtained from all patients to participate in this study after providing complete information about the disease, existing treatments, current treatment, and possible effects and complications. Patients are free to leave the study at any time. Also, the confidentiality of the patients' information was assured by the researcher.

## Conflicts of Interest

The authors declare no conflicts of interest.

## Data Availability

The data that support the findings of this study are available from the corresponding author upon reasonable request.
